# Glioblastoma Multiforme

**DOI:** 10.1155/2012/819304

**Published:** 2012-05-03

**Authors:** Jonas M. Sheehan, Robert Cavaliere, Elena Farace, Markus Bredel, Stuart H. Burri

**Affiliations:** ^1^Penn State Institute of the Neurosciences and Penn State Cancer Institute, Hershey, PA 17033, USA; ^2^The Ohio State University College of Medicine, Columbus, OH 43210, USA; ^3^Departments of Health Evaluation Sciences and Neurosurgery, Penn State College of Medicine/Penn State Milton S. Hershey Medical Center, Hershey, PA 17033, USA; ^4^Radiation Oncology Brain Tumor Laboratory, University of Alabama at Birmingham, Birmingham, AL 35233, USA; ^5^Division of Radiation Oncology, Levine Cancer Institute, Carolinas HealthCare System, Charlotte, NC 28203, USA

Despite recent advances in medicine, including the molecular and genetic advances in the field of oncology, glioblastoma remains one of the deadliest cancers in humans. Advances over the past 30 years have led to measurable but modest improvement in survival. New approaches to treatment, including vaccine therapy, based on sound laboratory and preclinical data, are encouraging, but the key to a substantial impact in patients with glioblastoma has remained elusive.

This special issue was undertaken in an effort to demonstrate some of the novel approaches to basic science, diagnosis, and treatment for patients with glioblastoma. The first manuscript discuses the use of a leading edge MR imaging technique, texture analysis, to differentiate glioblastoma from malignant glioneuronal tumors, highlighting advanced imaging techniques that should be further explored across brain tumors.

Basic science advances are also discussed. One article discusses the cause/effect role of leptin in glioblastoma, with a discussion regarding a potentially central role in tumor progression. In addition, E. A. El Habr et al. elucidate the clinical and prognostic role of the AKT-mTOR pathway in astrocytomas. These basic science developments are important as clinically relevant therapies are developed by logical extension from basic and translational knowledge.

Two other manuscripts focus on current and future trends in the management of glioblastoma. While the outcomes for patients with this deadly disease remain poor, it is our sincere hope that the work and effort demonstrated by our authors, and scores of researchers around the world, will lead to substantial advances in our understanding and management of glioblastoma.

*Jonas M. Sheehan* *Jonas M. Sheehan* *Robert Cavaliere* *Robert Cavaliere* *Elena Farace* *Elena Farace* *Markus Bredel* *Markus Bredel* *Stuart H. Burri* *Stuart H. Burri*

